# Treatment in severe acute pancreatitis – still a reason of debate

**Published:** 2013-12-25

**Authors:** D Popa

**Affiliations:** „Carol Davila” University of Medicine and Pharmacy Department of Surgery, University Emergency Hospital Bucharest, Romania

**Keywords:** Severe Acute Pancreatitis, Surgical therapy

## Abstract

Abstract

Surgical therapy in severe acute pancreatitis significantly evolved in the last 10 to 20 years. The aim was to present the experience of the First Surgery Clinic within the University Emergency Hospital Bucharest, in the management of severe acute pancreatitis, following major etiopathogenic, diagnostic and treatment aspects. Our study was retrospective, longitudinal and descriptive, including a seven years period, between 2004 and 2010. 42 patients diagnosed with severe acute pancreatitis have been admitted and operated. 25 were male, representing 59,52% and 17 female, respectively 40,47%. 55% of the patients were operated more than 11 days after the hospitalization, 25% were operated in the 4 to 10 days interval and 20% were operated in emergency conditions: immediate emergency (first 24 hours) or delayed emergency (24–72 hours). Mortality reported to the moment of surgery was 60% for the patients operated in the first 24 hours, 66,67% for the 24-72 hours operation interval, 30% for the patients operated between 4-10 days from the admission and 18,18% for operations performed after more than 11 days . Conclusion: mortality significantly decreased when the surgical moment was postponed by using intensive therapy, over 11 days from admission. We reconfirm the optimal temporization attitude of surgery, until the infection of necrosis or appearance of pancreatic abscess.

## Introduction

Surgical therapy in severe acute pancreatitis significantly evolved in the last 10 to 20 years.

Our study was retrospective, longitudinal and descriptive, including a seven years period, between 2004 and 2010. The aim was to present the experience of the First Surgery Clinic within the University Emergency Hospital Bucharest, in the management of severe acute pancreatitis, following major etiopathogenic, diagnostic and treatment aspects.

42 patients diagnosed with severe acute pancreatitis have been admitted and treated in our clinic between 2004-2010. Being a surgical department, only patients representing good candidates for surgical therapy were accepted, excluding the cases with mild forms of acute pancreatitis.

**Fig. 1:**
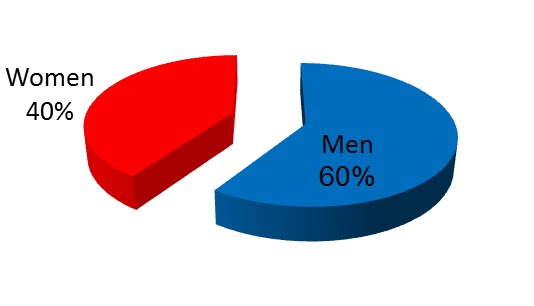
Gender distribution

 Out of the total number of 42 patients included in the study, 25 were male, representing 59,52% and 17 female, respectively 40,47%. We could observe the clear predominance of the masculine sex, explained mainly by the bigger incidence of etanolic etiology (**Fig. 1**).

**Fig. 2:**
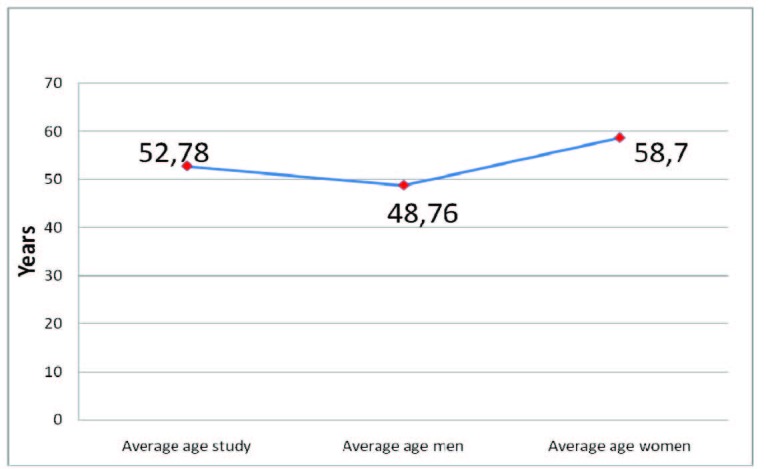
Age distribution

Mean age within the study was 52,78 years, with a minimum of 30 and a maximum of 88, respectively. Male mean age was 48,76 years, with a min of 30 and a max of 83. On the other hand, female mean age was 58,70 years, with a min of 31 years and a max of 88, respectively. There was a ten years difference between the genders, with an earlier debut in the male category (**Fig. 2**).

**Fig. 3:**
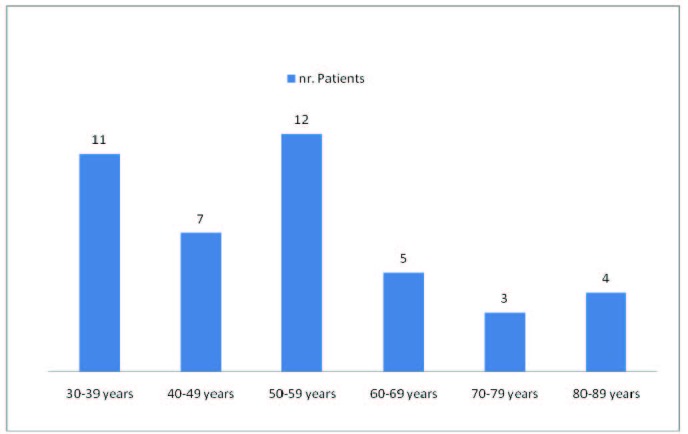
Age decade distribution

From the age decade’s point of view, we noticed a maximum number of patients in the sixth decade (50-59 years) – 12 patients, followed by the fourth decade (30-39 years) – 11 patients (**Fig. 3**).

**Fig. 4:**
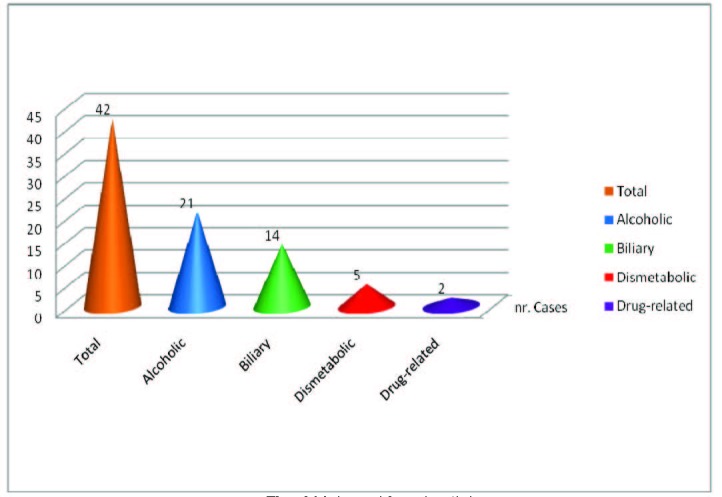
Male and female etiology

The etiology in pancreatitis group is represented by:

a) alcohol – 21 patients

b) lithiasis – 14 patients

c) dismetabolism – 5 cases

d) post-medication – 2 cases (**Fig. 4**).

Although in our clinic, diagnostic and therapeutic ERCP was available, there have been none SAP cases due to this invasive procedure during our study.

Alcohol represented the main etiology in male patients as well as biliary lithiasis was the major cause for the female gender.

**Fig. 5:**
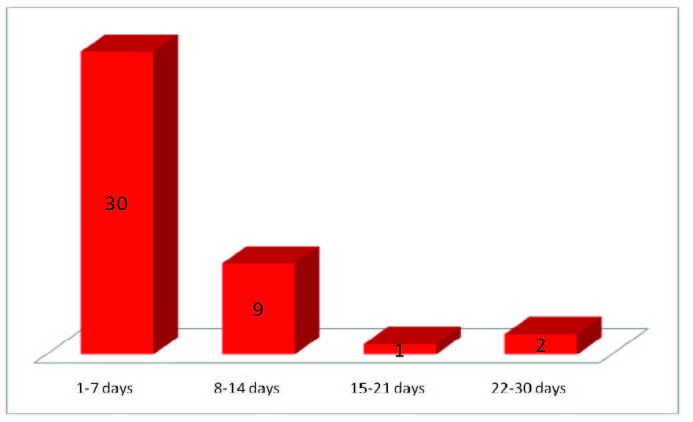
Mean number of days between the debut of the symptoms and the hospital admission

The mean number of days between the debut of the symptoms and the hospital admission was 5,94, with a minimum of zero to a maximum of thirty days from the onset of the symptoms. Out of the total number of 42 patients, six were transferred from other hospitals or other clinics within the University Hospital due to severe general evolution. Even in those cases, all the data from the hospital chart were properly analyzed, including the onset-admission time gap. We could observe the small number of days in the majority of the situations (71,42%), explainable by the severity of the symptoms, specific to SAP (**Fig. 5**).

Out of 42 patients, two patients were excluded due to the fact that they were not operated - those cases died before surgery. The remaining 40 patients had a mean period of 12,25 hospitalization days before surgery, with a maximum of 28 days and o minimum of zero, respectively.

55% of the patients were operated more than 11 days after the hospitalization, with a average number of 18,77 days between the admission to the surgical moment.

25% were operated in the 4 to 10 days interval (mean of 6,8 days time interval).

**Fig. 6:**
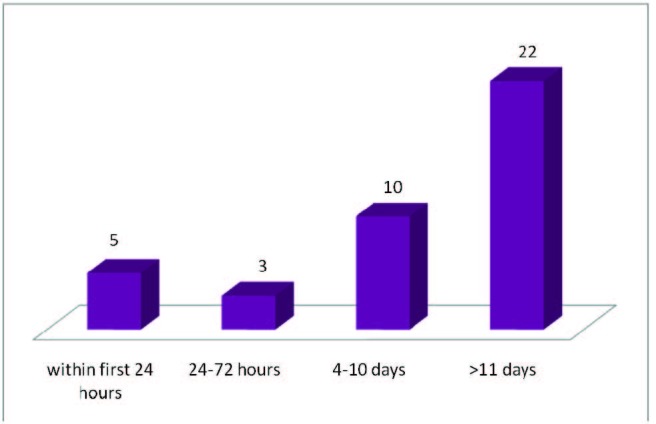
The mean time interval between admission to operation

20% were operated in emergency conditions: immediate emergency (first 24 hours) or delayed emergency (24–72 hours). In all these cases, the mean time interval was 1,12 days between admission to operation. There was either pancreatic ascites or abdominal compartment syndrome or imprecise diagnosis that forced us to operate on those patients on emergency settings (**Fig. 6**).

Mortality reported to the operation timing: for the deceased group (12 patients), the mean time interval admission-surgery was 8,75 days. Compared to the survival group we could observe that the same period was 13,75 days.

 From the 12 patients who died:

 - 3 patients have been operated in the first 24 hours due to acute surgical abdomen

 - 2 patients have been operated in the 24-72 hours period (delayed emergency).

- 3 patients had the surgical moment at 4-10 days from the admission 

- 4 patients were operated at more than 11 days from the admission.

**Fig. 7:**
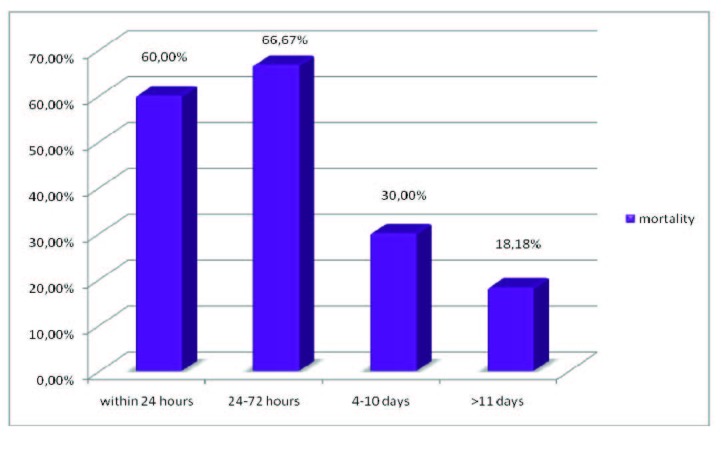
Mortality rates for patients

 Compared to the patients who survived (28 cases) and analyzing the same admission-operation intervals, we could observe the following:

 - 60% mortality rate for the surgery in the first 24 hours

 - 66,67% mortality rate for the 24-72 hours operation interval

 - 30% mortality for the patients operated between 4-10 days from the admission

 - 18,18% mortality for the patients operated more than 11 days from the hospitalization (**Fig. 7**)

 There was a correlation between the bacteriological findings and mortality. Out of the 40 patients who were operated, in seven situations the pancreatic necrosis proved to be sterile. For the rest of the cases, the lab results showed:

- Pseudomonas Aeruginosa in 10 cases

- Acinetobacter – 9 cases

- Klebsiella – 8 cases

- Escherichia Coli – 7 cases

- Staphylococcus Aureus – 5 cases

- Staphylococcus Epidermitis Coagulazo Pozitiv – 4 cases

These results also included the bacterial associations identified in some of the observations:

Acinetobacter, Klebsiella – 3 cases

 Pseudomonas Aeruginosa, Klebsiella – 2 cases

Pseudomonas Aeruginosa, Acinetobacter – 2 cases

**The importance of surgical moment:**

** a) the first 24 hours**

 - five patients included in this interval

- 12 operations in total with a median of 2,4 operations/patient

- Re-operations were performed in order to control the evolutive necrosis in the pancreas and in the retroperitoneal areas

- Median number of hospitalization days was 29,2

- Median blood units-2,6 anf frozen plasma units-2,4

 - The most frequent bacterial association was– E. Coli + Pseudomonas Aeruginosa and Staphylococcus Aureus + Klebsiella Pneumoniae

**b) 24-72 hours**

- three patients included in this interval

 - 12 operations in total with a median of 4 operations/patient

 - Re-operations were performed for haemoperitoneum (retro-colic area) 1 case, enteral fistula 1 case, diffuse peritonitis 2 cases and one pancreatic abscess

- Median number of hospitalization days was 52,33

 - Median blood units-5 and frozen plasma units-4,66

 - The most frequent bacterial culture was Acinetobacter, in two cases, followed by Escherichia Coli and Staphylococcus Aureus.

**c) 4-10 days**

 - ten patients included in this interval

-25 surgical procedures with a median of 2,5 operations/patient (decreasing)

-Re-operations were performed because of retroperitoneal evolutive necrosis (7 cases), pancreatic abscess (2 patients), haemoperitoneum (1 case), duodenal fistula (1 case), CBD stenosis (1 case), diffuse acute peritonitis (3 cases)

-Median number of hospitalization days was 61,3

-Median blood units-3 and frozen plasma units-3,5

-The most frequent bacterial culture was Pseudomonas Aeruginosa in five patients, followed by Acinetobacter, Klebsiella Pneumoniae, Escherichia Coli and Staphylococcus Aureus. Proteus was identified just in one situation

**d) Over 11 days**

 - 22 patients included in this interval

- 45 operations with a median of 2 procedures/patient

 - Re-operations were decided because of retroperitoneal necrosis (6 cases), pancreatic abscess (6 patients), haemoperitoneum (left retrocolic bleeding-1 case), duodenal fistula with suture (1 case), acute diffuse peritonitis (2 cases)

 - Median number of hospitalization days was 49,68

 - Median blood units-2,66 and frozen plasma units-3,41

- The most frequent bacterial cultures were Acinetobacter – 4 cases, Klebsiella Pneumoniae – 4 cases, Escherichia Coli – 4 cases, Staphylococcus Aureus – 4 patients and Pseudomonas Aeruginosa – 3 cases.

**Re-operations issue**

Our surgical attitude was to reoperate only when the conditions were imposing it, so, we did not adopt a programmed reoperation technique. Clinical suggestive signs were represented by unfavorable evolution, with febrile and persistent septic signs. The images that conducted us to the operation decision were the abdominal ultrasound and C.T. scan. These showed the existence of collections in the lesser sack, Douglas pouch, subphrenic spaces or between the bowel loops and the re-appearance of necrotic areas in the pancreas or retroperitoneal spaces.

Out of 40 operated patients who were included in our study, we had the following outcomes:

- 54 surgical reinterventions, with a maximum of seven operations for one patient who also died

- 33 reinterventions for the control of the pancreatic and retroperitoneal necrosis.

- Three reinterventions for bleeding: two retrocolic on the left side and one pancreatic.

- Two reinterventions for fistulas: one duodenal and one enteral.

 - One reintervention for extensive stenosis of the common bile duct (CBD).

 - Six reoperations for abdominal abscesses.

- Nine operations for acute diffuse peritonitis.

 Out of the 40 patients, 27 were multiply operated with 10 deaths. We could observe that, out of a total of 14 deaths, the majority was represented by multiple operated cases.

 In the same time, from the total of 27 cases multiply operated, in 14 situations, open abdomen with mesh technique was used.

**Complications issue**: being an evolving disease with a reserved prognosis, SAP is characterized by a large number of complications, both general and local.

General complications were represented by:

- Acute renal failure – six cases (14,28%).

- Pleurisy – 21 cases, out of which five needed surgical drainage (11,9%).

 - Hemorrhagic gastritis – three cases (7,14%).

- Pneumonia – two cases (4,76%).

Local complications were represented by:

- Bleeding – in the peritoneal cavity, on the debridement areas or pancreatic space. Three cases (7,14%): two cases from the left retrocolic space and one case from pancreatic zone.

- Digestive Fistulas – severe complication in the postoperative evolution of SAP. First of all, the treatment has to be supportive and conservative. If the fistula flow is big, surgical treatment is needed, consisting in suturing and vicinity drainage. We had two cases with fistulas (4,76%), both of the patients survived:

 - One duodenal fistula;

 - One enteral fistula.

- CBD stenosis was present in one observation– 2,38% (extensive stenosis until the junction of the two hepatic ducts). Diagnosis was made by ERCP and the patient was considered to be over the therapeutical resources.

- Residual abdominal abscesses– were associated in the evolution of six patients (14,28%), two right subphrenic abscesses and four between small bowel loops. Treatment in those cases was strictly surgical.

- Pancreatic fistula – was found in four cases. It appeared as a complication after extensive pancreatic debridement and their therapy was conservative.

- Pancreatic abscess (PA) – was found in seven cases (6 male patients). Etiology was etanolic in four cases and biliary in the other three. Median time between the debut to the surgery was 22 days with a minimum of 12 days and a maximum of 34. Median number of operations was four with a maximum of seven, for a patient that also died.

## Discussion, Conclusions

 - when comparing the demographic structure with the Romanian and international literature, we found a great variability according to the country, region and the size of studied population. Normally, female patients are leading where biliary etiology is predominant; it is all the other way for alcoholic etiology [**1**-**3**].

 - our study group followed the characteristics of previous Romanian studies with a higher incidence for male patients

 - there was a predominance of biliary etiology for SAP in Romanian literature. In our analysis alcoholic etiology was predominant, although sex distribution was similar with the other Romanian published papers [**3**,**4**].

 - currently, all the authors think that best time for operation in SAP is when pancreatic necrosis has been defined or when septic complications appear. The third week of evolution is the moment when these conditions are accomplished [**5**]. In the same time, a consensus has been reached regarding the moment when necrosis becomes infected: between the second and the third week of evolution.

 - the International Association of Pancreatology (IAP) Guide stands that it is preferable to operate between the 15-28th day (weeks 3-4) from the debut of the disease (not from the admission!!) [**6**]. Nevertheless, an European survey showed that there is no consensus in that direction, almost half of the surgeons, 43%, decided to operate in the first 14 days of evolution and only less than a quarter (29%) decided to wait for 21 days [**7**]. Analyzing the Romanian literature, Cochior and Constantinoiu published a study in Chirurgia 2010 about the importance of the surgical moment in SAP, with a median of 19 days from the debut of the disease to the operation [**8**].

- the biggest mortality rate occurred when emergency operations appeared. 

 - mortality significantly decreased when the surgical moment was postponed by using intensive therapy, over 11 days from admission.

 - in conclusion, in our analysis, we reconfirmed the optimal temporization attitude of surgery, until the infection of necrosis or appearance of pancreatic abscess.
